# The use of teat disinfectants and milking machine cleaning products in commercial Holstein-Friesian farms

**DOI:** 10.3389/fvets.2022.956843

**Published:** 2022-10-19

**Authors:** László Ózsvári, Dorottya Ivanyos

**Affiliations:** Department of Veterinary Forensics and Economics, University of Veterinary Medicine Budapest, Budapest, Hungary

**Keywords:** dairy, udder health, mastitis, milk quality, teat disinfectants, milking machine cleaning

## Abstract

The aim of our study was to survey and analyze the use of pre- and post-milking teat disinfectants and milking machine cleaning products in large commercial Holstein-Friesian farms. A total of 43 Hungarian dairy farms with 31,430 cows with an average herd size of 731 cows were surveyed in 2014 by using a questionnaire *via* personal interviews. In the statistical analysis, we used ANOVA models and Tukey's multiple comparison method. Furthermore, seven in-depth individual interviews were conducted with farm managers. The results showed that 83.7% of the farms used different pre-milking disinfection methods (65.1% teat dips) and all of them applied post-milking disinfection. In the herds, chlorhexidine (42.9%) and other chlorine (21.4%) compounds were the most widely used active ingredients in the pre-milking disinfection, while iodine in the post-milking disinfection (53.8%). Lactic acid was ranked second in both disinfections (25.0 vs. 41.0%). In post-milking teat disinfection, the use of iodine and lactic acid combined with other active ingredients showed a significant relationship with SCC (*p* = 0.0454; *p* = 0.0113). In the milking machine cleaning process, the most frequently used active ingredients were sodium hypochlorite (80.0%) and sodium hydroxide (60.0%) as caustic detergents, while phosphoric acid (81.3%) as an acidic product. A significant relationship was found between the use of phosphoric acid combined with nitric acid, and the use of a combination of phosphoric acid, nitric acid, and organic acid and SCC (*p* = 0.0483; *p* = 0.0477). For farm decision-makers, the most decisive factor in the procurement of teat disinfectants was the active ingredient (3.4 on a scale of 1 to 10 where 1 was the most important), while regarding milking machine cleaning products the price (3.2).

## Introduction

Mastitis is one of the most frequent diseases of dairy cows and has well-recognized detrimental effects on animal wellbeing and dairy farm profitability (1). According to the National Mastitis Council (NMC) Recommended Mastitis Control Program, routine application of pre- and post-milking teat disinfectants during each milking is highly recommended to prevent new intramammary infections. Teat disinfectants should meet several requirements: (1) have proven germicidal efficacy, (2) prevent new intramammary infections, (3) maintain optimal teat condition and promote lesion healing, (4) not irritate the cow or the user, and (5) leave no residues in milk that could affect human health (2). Various teat cleaning disinfectants, including iodophor solution, iodine-based gel, sodium hypochlorite, dodecyl benzene sulfonic acid, chlorine, chlorhexidine, phenolic compounds, alcohol, and guava leaf extract, are used for pre-milking teat dipping (3). Iodine-based teat products are most used to disinfect teats before and after milking (4).

The milking machine cleaning has an important role in the reduction of bacterial numbers in milk (5). Cleaning and sanitation of milking equipment is a combination of chemical, thermal, and physical processes which need a minimum reaction time to be effective (6). The typical automatic cleaning process can be divided into three different main phases: pre-rinse, washing phase, and post-rinse. The pre-rinse phase is essential to remove most milk residues. During the washing phase, alkaline or acid detergents should be used. The alkaline detergent helps to remove organic deposits, such as milk protein and fat. Acid detergent is used periodically to remove mineral deposits from water and milk (7). A high proportion (67%) of liquid products used for cleaning and disinfection of Irish milking machines contain sodium hypochlorite, but some milk processors are now recommending the use of non-chlorine liquid detergent cleaning products such as sodium hydroxide or acids (5).

It is well-known that mastitis and milk quality are associated with teat disinfectants and milking machine cleaning products, but several practical aspects of their use, particularly regarding the milking machine cleaning practices, and their statistically confirmed impact on milk production in dairy cattle herds can contribute to the current knowledge in this field. Therefore, the aim of our study was to survey the practical use of pre- and post-milking teat disinfectants and milking machine cleaning products and analyze the associations between disinfection and cleaning practices, herd size, and milk production parameters in commercial Holstein-Friesian farms.

## Materials and methods

### Study design

The survey was drafted to define the use of teat disinfectants and milking machine cleaning products in commercial Holstein-Friesian farms and the views of farm managers on teat disinfection and cleaning practices. The drafted survey was reviewed by farm managers (*n* = 2), dairy cattle veterinary practitioners (*n* = 3), academic professionals (*n* = 3), and veterinary and animal science Ph.D. students (*n* = 3) to receive feedback on content. Based on collected feedback, revisions were made before the survey was sent to potential respondents. This study used a mixed-method approach, which combines the collection and analysis of quantitative and qualitative data. In the first part of this work, we collected data about the total number of cows, type of milking system and parlor, number of daily milkings, milk production parameters [lactation milk yield, somatic cell count (SCC), percentage share of marketed milk, and days in milk (DIM)], and we also surveyed the active ingredients of the used pre- and post-milking teat disinfectants and milking machine cleaning products (detergents and disinfectants), and their applications (e.g., disinfection and cleaning processes, the concentration of substances). The key factors in the procurement of teat disinfectants and milking machine cleaning products were also surveyed by evaluating their different characteristics on a scale of 1 to 10 (“1” for the most important, “10” for the least important factor). In the second part of the in-depth survey, structured individual interviews were conducted with dairy cattle farm managers. We used a questionnaire with open-ended questions that allowed the participants to convey their views on the aforementioned teat disinfection and milking machine cleaning practices.

### Data collection

Commercial Holstein-Friesian farms were included in this survey based on the following criteria: use of computerized on-farm records, participation in milk recording, and willingness to provide data to the authors. A total of 43 Hungarian dairy farms were surveyed between September and October 2014 by using a questionnaire *via* personal interviews with farm managers (*n* = 21; 48.8%), veterinarians (*n* = 14; 32.6%), shift supervisors (*n* = 5; 11.6%), or division heads (*n* = 3; 7.0%), who had access to farm records and were familiar with the milking procedures in the studied dairy units. Furthermore, we had in-depth, structured individual interviews with seven farm managers (out of the 21). The participants took part in the survey voluntarily and remained anonymous. Each participant was required to sign a written consent before they began the survey. Each questionnaire has been coded to detect inaccuracies in data entry. The obtained data were processed in MS Excel (Microsoft Corporation, Redmond, WA, USA).

A total of 31,430 cows were kept on the 43 farms, which corresponded to 17.8% of the 176,753 Hungarian dairy cow population on 458 performance-tested farms according to the official statistical data during the time of the survey (8, 9). The smallest surveyed farm had 56 cows, whereas the largest had 2,500; the average herd size was 731 ± 508 (milking + dry cows). All of the seven regions of Hungary were covered in the survey (min. 3 and max. 14 dairy farms per region were involved). The seven, individually interviewed farm managers represented a total of 6,130 cows with an average herd size of 876 ± 779 (*n* = 7; min. 300; max. 2500).

Total annual milk production per farm was 6,712,655 kg (*n* = 43; min. 321,484 kg; max. 22,522,000 kg), of which 96.8% was marketed (min. 90.0%, max. 99.3%). The average lactation milk yield was 9,716 kg (*n* = 41; min. 5,409 kg; max. 11,915 kg), average milk fat content was 3.7% (*n* = 41; min. 2.97%; max. 4.16%), average milk protein content was 3.3% (*n* = 41; min. 3.17%; max. 4.2%), and average SCC was 419,000 (*n* = 38; min. 188,000; max. 936,000), respectively. The average length of lactation was 373 days (*n* = 26; min. 310 days; max. 545 days).

More than half of the milking parlors had a herringbone design (57.8%), followed by parallel (20.0%), rotary (17.8%), and polygon (4.4%) milking systems. The average age of the milking systems was 11.7 years (*n* = 41; min. 1 year; max. 28 years). The cows were mostly milked twice a day (62.8%), but 41.9% of the farms milked the cows three times and 4.7% four times a day. On four farms different cow groups were milked differently (usually cows were milked more frequently until 30 DIM). The vast majority of the farms (85.4%) used traditional elastic teat liners and 14.6% silicone ones. The teat cups were disconnected automatically on all farms, except for the smallest one.

All herds (*n* = 43) were free from tuberculosis, brucellosis, and bovine leukosis, but 34.9% of the farms were also free from either IBR (25.6%), BVD (4.7%) or the five diseases (4.7%). The diseased cows (e.g., clinical mastitis cases) were kept in separate hospital barns on 59.5% of the surveyed farms (*n* = 42). In the other herds, they were isolated within the maternity barn.

### Statistical analysis

The surveyed farms represented all farm sizes, milking systems, milking parlor types, and geographical regions in Hungary. Teat disinfection and milking machine cleaning practice outcome measures were analyzed with ANOVA models. All models included the herd size (1–400, 401–800, and >800 cows), milking parlor type (herringbone, parallel, rotary, polygon), and number of daily milkings (two times, more than two times) as explanatory variables. Consequently, bias caused by data imbalance related to these variables was eliminated from the resulting estimates. For each pre- and post-milking teat disinfection and milking machine cleaning practice outcome, the basic model included only the three main management variables listed above. Next, each management explanatory variable was added to the basic model one by one separately (Tables 1, 2). The normality of the residuals (the difference between the raw data and the model prediction) is checked by quantile comparison plots (QQ-plot), and no deviation from normality was found. Differences between the means of the outcome variables in the layers of the explanatory variables were evaluated by Tukey's multiple comparison method applying the R package multcomp. Statistical analyses were performed in R version 4.1.2 (10). The level of significance was set to 0.05. To support confirmatory analysis with newly collected data, in cases of marginally significant differences (i.e., when the *p*-values were >0.05 and < 0.1), we carried out power analysis assuming equal sample sizes in the groups compared. The calculations were carried out applying the method of Cohen (11), implemented in the package pwr of R version 4.1.2 (10). The effect sizes (i.e., the ratios of mean differences to residual standard deviations) were set to 0.88, which is the smallest effect size obtained from the ANOVA models in this paper. The required power was set to 0.8, and the family-wise error rate related to the multiple comparisons of group means was set to 0.05. We applied Bonferroni corrections to adjust *p*-values in multiple comparisons. In summary, the power analysis revealed that the smallest eligible sample size for the new balanced study was 23.

**Table 1:** The analyzed teat disinfection explanatory variables.

**Variable**
Use of pre-milking disinfectant	Yes
	No
Component number of a pre-milking	One
teat disinfectant
	Two
	Use of impregnated paper
Active ingredient of pre-milking	Alcohol
teat disinfectant
	Chlorine
	Chlorine/alcohol
	Chlorine/iodine
	Chlorine/lactic acid
	Lactic acid
	Other acid
Using chlorine as a pre-milking teat disinfectant	Yes
	No
Using lactic acid as a pre-milking teat disinfectant	Yes
	No
Using alcohol as a pre-milking teat disinfectant	Yes
	No
Component number of a post-milking	One
teat disinfectant
	Two
Active ingredient of a post-milking disinfectant	Chlorine
	Iodine
	Iodine/chlorine
	Iodine/lactic acid
	Iodine/lactic acid/chlorine
	Iodine/other
	Lactic acid
	Lactic acid/chlorine
	Lactic acid/other
	Other
Use of iodine as a post-milking disinfectant	Yes
	No
Use of lactic acid as a post-milking disinfectant	Yes
	No

**Table 2:** The analyzed milking machine cleaning explanatory variables.

**Variable**
Phases of cleaning	3
	5
	Both
Rinsing	After cleaning
	Before cleaning
Acid concentration	1%
	Not 1%
Caustic concentration	1%
	Not 1%
Disinfectant concentration	≤ 1%
	>1%
Daily cleaning	After every milking
	Not after every milking
Number of weekly acid cleanings	1–3.5
	7–21
Number of yearly manual cleanings	0–12
	24–52
Caustic ingredients	Sodium hydroxide
	Sodium hydroxide/sodium hypochlorite
	Sodium hypochlorite
	Other
Acid ingredients	Nitric acid
	Organic acids
	Phosphoric acid
	Phosphoric acid/nitric acid
	Phosphoric acid/nitric acid/organic acid
	Phosphoric acid/sulfuric acid
Use of phosphoric acid	Yes
	No
Use of nitric acid	Yes
	No
Use of sulphuric acid	Yes
	No
Use of disinfectants	Yes
	No
Disinfectant ingredients	Hydrogen peroxide
	Peracetic acid
	Peracetic acid/hydrogen peroxide

## Results

There was no significant relationship between the herd size and the milking parlor type, and the lactation milk yield and SCC (*p* ≥ 0.108 and *p* ≥ 0.192). The percentage share of marketed milk tended to be higher in parallel parlors vs. herringbone (*p* = 0.0772) and DIM tended to be higher in herringbone parlors vs. polygon (*p* = 0.0729), and in herds with >800 cows vs. 1–400 cows (*p* = 0.0892) by Tukey's multiple comparison method. A significant relationship was found between the number of daily milkings and lactation milk yield (*p* = 0.0182).

### Pre-milking teat disinfectants

Vast majority of the surveyed farms (83.7%) used different pre-milking teat disinfection procedures, 65.1% used disinfectant teat dips, 14.0% disinfectant wash or foaming, and 4.7% impregnated papers (Supplementary Table 1). No significant association was found between the use of pre-milking disinfection and the studied milk production parameters (lactation milk yield, percentage share of marketed milk, DIM, SCC; *p* ≥ 0.3356). Six out of seven (85.7%) personally interviewed farm managers said that pre-milking teat disinfection should always be applied and four out of seven (57.1%) considered disinfectant dip or foaming disinfectant wash to be the ideal method.

Out of the farms applying pre-milking teat disinfection 72.2% used one-component pre-milking disinfectants exclusively, 8.3% both one- and two-component disinfectants, 13.9% only two-component products, while two large dairies (5.6%) used disinfectant impregnated paper (Supplementary Table 2). The use of one component pre-milking disinfectant and the use of impregnated paper showed significant association with DIM (*p* = 0.0125; *p* = 0.0376) and the use of two-component pre-milking disinfectant tended to associate with SCC and DIM (*p* = 0.0865; *p* = 0.0716). All seven personally interviewed farm managers preferred one-component pre-milking disinfectants, because of their practical use primarily (71.4%), that is, they exclude the possibility of human errors, but the longer shelf life and sales discounts were also mentioned (14.3% each).

**Figure 1:**
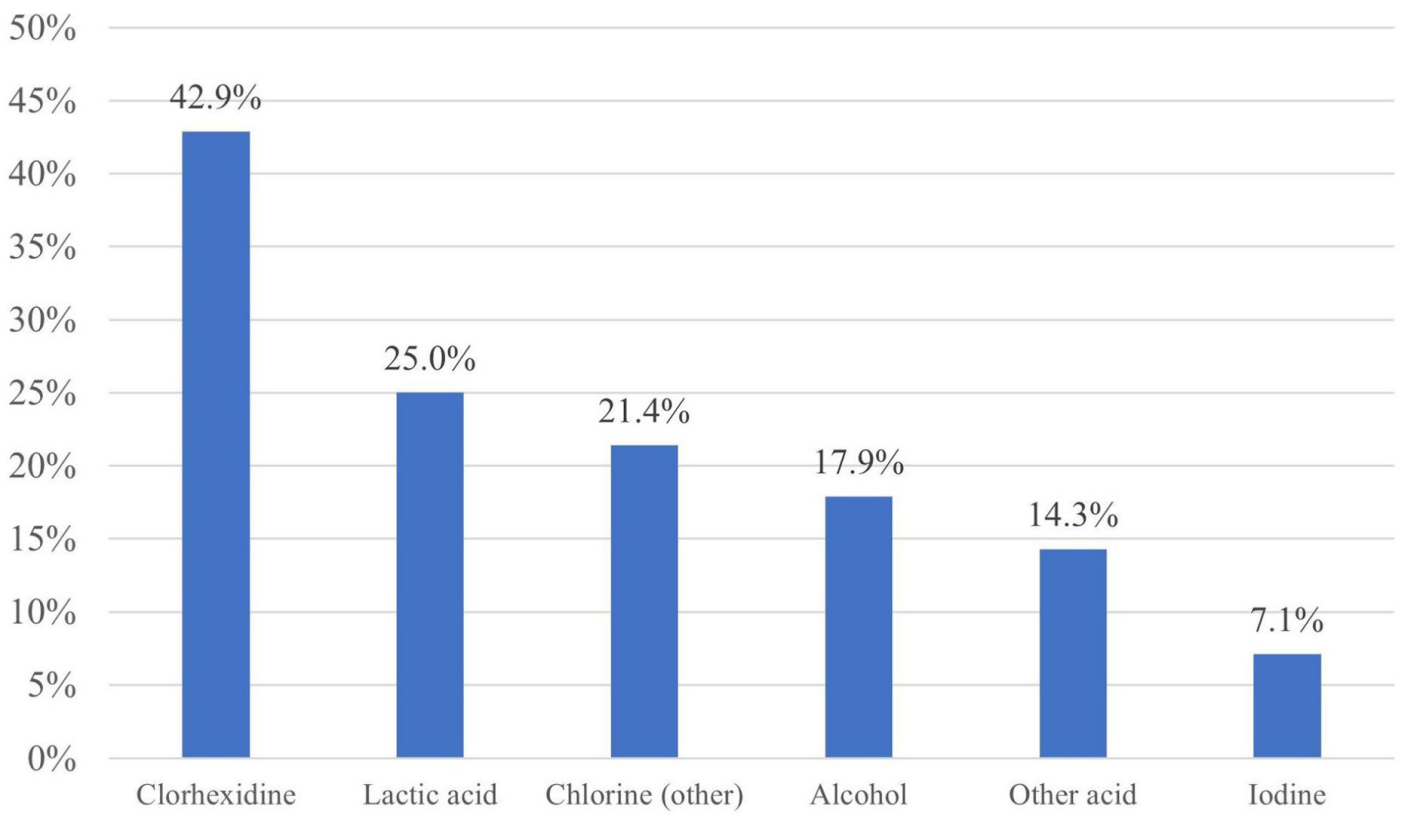
Active ingredients of the pre-milking teat disinfectants (*n* = 28).

The most used active ingredient of the pre-milking disinfectants was chlorhexidine (42.9%), followed by lactic acid (25.0%) and other compounds of chlorine (chlorine dioxide and triclosan together: 21.4%). The pre-milking disinfectants containing compounds of chlorine were used in almost two-thirds (64.3%) of the commercial dairy units, while disinfectants with iodine were the least preferred products (7.1%) (Figure 1). No significant association was found between the active ingredients and the studied milk production parameters (*p* ≥ 0.1535) (Tables 3–6). Several farms used multiple pre-milking teat disinfectants at the same time.

**Table 3:** Relationships between somatic cell count (SCC/ml) and pre- and post-milking disinfectants.

**Variables**	**Layers**	**Mean**	**SD**	***n***	***P*-value**
Active ingredient pre-milking disinfectant	Alcohol	472.333	111.114	3	
	Chlorine	481.000	190.711	12	0.8680
	Chlorine/alcohol	386.000		1	0.9295
	Chlorine/iodine	365.000		1	0.4908
	Chlorine/lactic acid	390.000		1	0.5497
	Lactic acid	459.800	217.520	5	0.6347
	Other acids	220.000		1	0.3745
Using chlorine as a pre-milking disinfectant	No	437.333	182.863	9	
	Yes	460.867	174.184	15	0.5010
Using lactic acid as a pre-milking disinfectant	No	453.333	171.810	18	
	Yes	448.167	196.695	6	0.6604
Using alcohol as a pre-milking disinfectant	No	452.300	187.096	20	
	Yes	450.750	100.470	4	0.6088
Active ingredient of a post-milking disinfectant	Chlorine	424.200	101.726	5	
	Iodine	408.889	183.767	9	0.0454*
	Iodine/chlorine	582.500	307.592	2	0.3956
	Iodine/lactic acid	566.667	202.073	3	0.1801
	Iodine/lactic acid/chlorine	395.000		1	0.6219
	Iodine/other	550.000		1	0.7513
	Lactic acid	429.500	183.903	8	0.9436
	Lactic acid/chlorine	250.000	42.426	2	0.1358
	Lactic acid/other	643.000	414.365	2	0.0113*
	Other	350.000		1	0.2030
Using iodine as a post-milking disinfectant	No	444.333	184.796	18	
	Yes	479.722	183.276	18	0.1841
Using lactic acid as a post-milking disinfectant	No	491.095	201.472	21	
	Yes	421.333	148.430	15	0.2671

^*^Significant relationships (p < 0.05).

**Table 4:** Relationships between lactation milk yield (kg) and pre- and post-milking disinfectants.

**Variables**	**Layers**	**Mean**	**SD**	***n***	***P*-value**
Active ingredient pre-milking disinfectant	Alcohol	10.284	415	4	
	Chlorine	9.551	1.056	11	0.1535
	Chlorine/alcohol	9.853		1	0.1896
	Chlorine/iodine	10.200		1	0.3134
	Chlorine/lactic acid	10.300		1	0.3558
	Lactic acid	9.059	921	5	0.4425
	Other acids	8.483	81	2	0.3158
Using chlorine as a pre-milking disinfectant	No	9.400	964	11	
	Yes	9.833	1.056	16	0.3409
Using lactic acid as a pre-milking disinfectant	No	9.757	1.060	20	
	Yes	9.371	925	7	0.9721
Using alcohol as a pre-milking disinfectant	No	9.512	1.108	21	
	Yes	10.165	374	6	0.4245
Active ingredient of a post-milking disinfectant	Chlorine	8.737	1.821	5	
	Iodine	9.974	1.075	11	0.9038
	Iodine/chlorine	8.900	1.838	2	0.5961
	Iodine/lactic acid	8.020	2.321	3	0.8750
	Iodine/lactic acid/chlorine	10.200		1	0.9197
	Iodine/other	8.400		1	0.2156
	Lactic acid	9.112	1.316	8	0.5454
	Lactic acid/chlorine	9.210	948	2	0.8105
	Lactic acid/other	9.356	786	2	0.9439
	Other	10.600		1	0.2637
Using iodine as a post-milking disinfectant	No	8.945	1.369	18	
	Yes	9.603	1.308	21	0.7381
Using lactic acid as a post-milking disinfectant	No	9.358	1.435	23	
	Yes	9.215	1.285	16	0.4736

**Table 5:** Relationships between the share of marketed milk (%) and pre- and post-milking disinfectants.

**Variables**	**Layers**	**Mean**	**SD**	***n***	***P*-value**
Active ingredient pre-milking disinfectant	Alcohol	96.3	1.8	4	
	Chlorine	96.8	1.8	11	0.641
	Chlorine/alcohol	93.5		1	0.301
	Chlorine/iodine	98.8		1	0.260
	Chlorine/lactic acid	ND		0	
	Lactic acid	97.7	1.3	5	0.407
	Other acids	97.4	1.2	2	0.761
Using chlorine as a pre-milking disinfectant	No	97.1	1.5	11	
	Yes	96.8	1.9	15	0.733
Using lactic acid as a pre-milking disinfectant	No	96.6	1.8	20	
	Yes	97.9	1.3	6	0.184
Using alcohol as a pre-milking disinfectant	No	97.1	1.6	20	
	Yes	96.3	2.2	6	0.444
Active ingredient of a post-milking disinfectant	Chlorine	96.4	2.0	4	
	Iodine	97.1	1.5	13	0.675
	Iodine/chlorine	96.0	3.8	2	0.553
	Iodine/lactic acid	95.9	5.1	3	0.743
	Iodine/lactic acid/chlorine	98.0		1	0.970
	Iodine/other	ND		0	0.313
	Lactic acid	97.1	1.1	6	0.940
	Lactic acid/chlorine	98.1	0.1	2	0.341
	Lactic acid/other	96.9		1	0.657
	Other	96.5		1	0.889
Using iodine as a post-milking disinfectant	No	96.8	2.1	17	
	Yes	96.7	2.4	17	0.880
Using lactic acid as a post-milking disinfectant	No	96.3	2.8	18	
	Yes	97.2	1.6	16	0.403

ND, no data.

**Table 6:** Relationships between Days in Milk (days) and pre- and post-milking disinfectants.

**Variables**	**Layers**	**Mean**	**SD**	***n***	***P*-value**
Active ingredient of a pre-milking disinfectant	Alcohol	329	16	2	
	Chlorine	382	46	8	0.3464
	Chlorine/alcohol	394		1	0.1601
	Chlorine/iodine	350		1	0.3417
	Chlorine/lactic acid	ND			
	Lactic acid	393	34	4	0.1584
	Other acids	367		1	0.2278
Using chlorine as a pre-milking disinfectant	No	371	39	7	
	Yes	380	42	10	0.6770
Using lactic acid as a pre-milking disinfectant	No	371	42	13	
	Yes	393	34	4	0.6017
Using alcohol as a pre-milking disinfectant	No	382	39	14	
	Yes	350	40	3	0.6888
Active ingredient of a post-milking disinfectant	Chlorine	361	38	5	
	Iodine	351	22	5	0.2751
	Iodine/chlorine	360	14	2	0.9563
	Iodine/lactic acid	339	26	2	0.9430
	Iodine/lactic acid/chlorine	450		1	0.0808
	Iodine/other	ND		0	
	Lactic acid	399	24	4	0.3343
	Lactic acid/chlorine	411	62	2	0.4413
	Lactic acid/other	405	35	2	0.3813
	Other	452		1	0.1234
Using iodine as a post-milking disinfectant	No	385	44	11	
	Yes	368	42	14	0.9860
Using lactic acid as a post-milking disinfectant	No	370	42	14	
	Yes	383	44	11	0.5039

ND, no data.

Three out of the seven interviewed farm managers (42.9%) considered chlorhexidine to be the optimal active ingredient in pre-milking disinfection, although another three managers (42.9%) considered iodine to be equally good. One manager (14.3%) also mentioned lactic acid as an optimal active ingredient. Six out of seven farm managers (85.7%) named efficacy as the major reason for choosing a certain active ingredient for pre-milking disinfection.

### Post-milking teat disinfectants

All the surveyed farms applied post-milking teat disinfection and 67.5% of the farms exclusively used one-component post-milking disinfectants, 27.5% two-component products alone, and a further 5.0% both (Supplementary Table 3). No significant relationship was found between the component number of the post-milking disinfectant and the studied milk production parameters (*p* ≥ 0.1300). Six out of seven interviewed farm managers (85.7%) preferred one-component post-milking disinfectants, because of their practical use primarily (57.1%), but the efficacy, sales discount, and longer shelf life were also mentioned (14.3% each).

**Figure 2:**
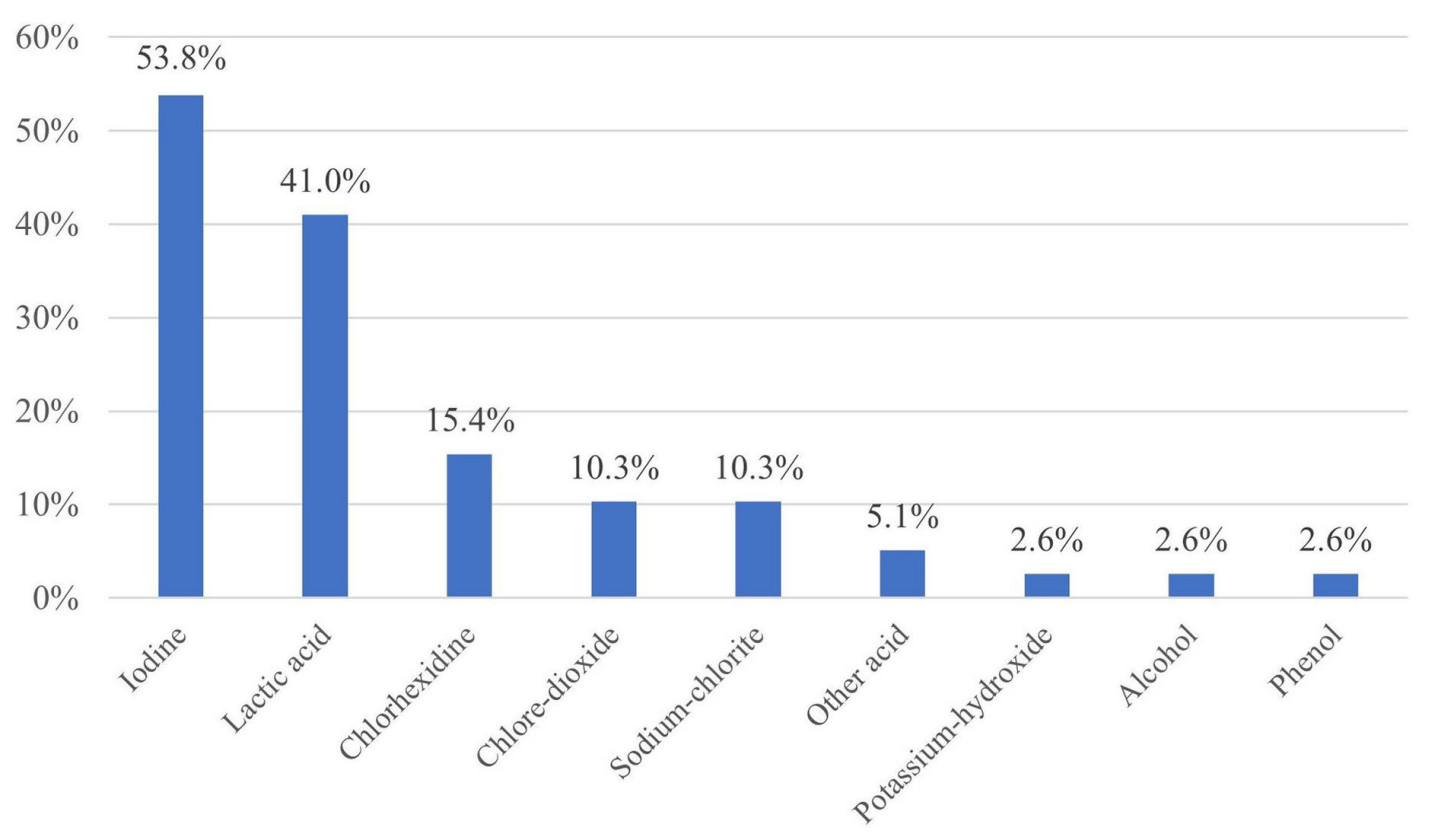
Active ingredients of the post-milking teat disinfectants (*n* = 39).

Iodine was the most used active ingredient in post-milking disinfection on the farms (53.8%), followed by lactic acid (41.0%) and various compounds of chlorine (chlorhexidine, chlorine dioxide and sodium chlorite) with a total share of 36.0% (Figure 2). The use of iodine and lactic acid combined with other active ingredients showed a significant relationship with SCC (*p* = 0.0454; *p* = 0.0113) (Tables 3–6). Several farms used multiple post-milking teat disinfectants at the same time. Six out of seven individually interviewed farm managers (85.7%) considered iodine as the optimal active ingredient in post-milking disinfection, although two managers (28.6%) also named lactic acid and chlorhexidine. The major reason for the choice of an active ingredient was the efficacy (100%), but one farm manager (14.3%) underlined the effectiveness against *Staphylococcus aureus* and another one (14.3%) mentioned the importance of skin integrity, referring to the fact that iodine dries the skin.

**Figure 3:**
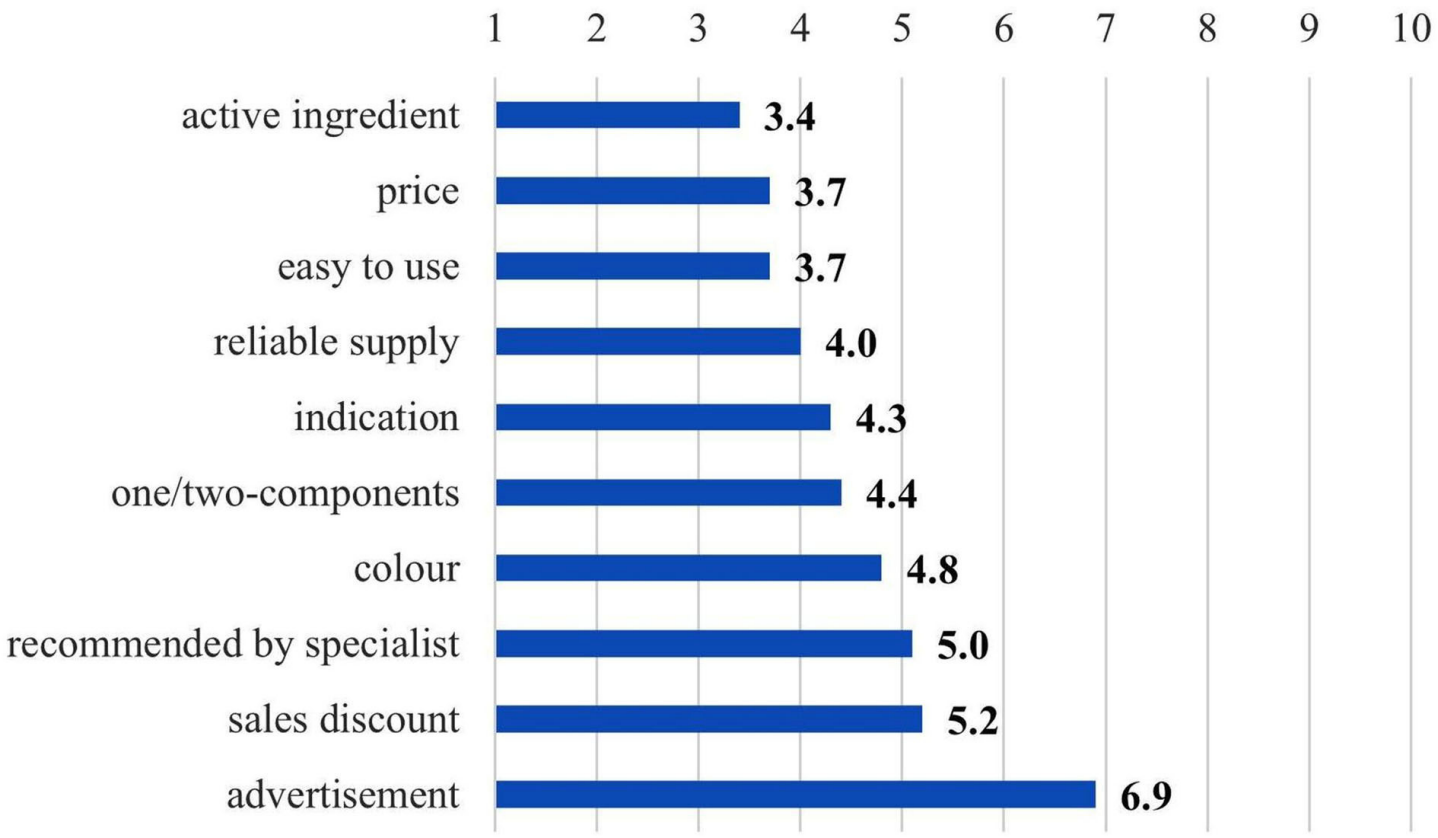
Importance of different procurement factors for pre- and post-milking disinfectants (*n* = 43).

The key factors in the procurement of teat disinfectants were also surveyed, the farm decision-makers evaluated the different characteristics of pre- and post-milking disinfectants on a scale of 1 to 10 (“1” for the most important, “10” for the least important factor). Figure 3 shows that the active ingredient was the most decisive procurement factor for a teat disinfectant, followed by price and ease of use. The advertisement was considered as the least important factor in the procurement, and sales discounts were not that important either.

The seven personal interviews confirmed the results of the survey, since the most important teat disinfectant characteristic for all seven farm managers was efficacy, followed by color of the product (85.7%), good value/price ratio (71.4%), reliable supply (57.1%), no skin drying effect (42.9%), and proper teat skin coverage (28.6%). The solution color makes it easy for the farm managers to check if the disinfectant was applied correctly. Two farm managers (28.6%) underlined the importance of skin coverage and the ability of the product to stay on the skin, that is, the disinfectant should not drip off, it should stay on the teat for a long enough time so that it can take full effect. Other factors, namely, number of milking sessions, package size, easy to use, small per-unit cost, viscosity and distributor's recommendation were mentioned once by the farm managers (14.3% each).

### Milking machine cleaning products (detergents and disinfectants)

About 59.5% of the surveyed farms used a 5-phase cleaning system (pre-rinse; caustic wash; rinse; disinfection; final rinse), 35.7% applied three-phase systems (pre-rinse; sanitizing wash including cleaning and disinfection; final rinse), and 4.8% used both systems depending on timing and duration of the milking session (Supplementary Table 4). We found no significant relationship between the type of cleaning and the studied milk production parameters (*p* ≥ 0.1522). Four out of seven interviewed farm managers (57.1%) considered the three-phase cleaning systems better than the 5-phase ones. 74.4% of the farms performed the final rinsing cycle immediately after disinfection and 25.6% directly before the next milking session (*n* = 39). The time of the final rising tended to associate with SCC (*p* = 0.0634). All the interviewed farm managers considered rinsing to be optimal directly after disinfection.

Milking machines were cleaned twice a day in 73.8% of the farms, three times a day in 21.4%, but only once a day in 4.8%, respectively (Supplementary Table 5). It can be stated that the milking machinery was not cleaned after every milking on many farms (especially in the case of three milking sessions a day), despite the fact, that all seven interviewed farm managers said that cleaning should be performed after each milking optimally. There was no significant relationship between daily cleaning and the studied milk production parameters (*p* ≥ 0.1254).

The number of acid descaling washes per week revealed a very diverse picture: it ranged from once a week (in 11.9% of the farms) to 21 times a week (11.9%), that is, after every milking, but in most cases, it was performed daily (26.2%) or 2 (23.8%) to 3 (16.7%) times per week (Supplementary Table 6). There was no significant relationship between the number of weekly acid cleaning and the studied milk production parameters (*p* ≥ 0.2588). Even the opinions of the interviewed farm managers on the optimal number of acid descaling washes per week differed: three out of seven (42.9%) said an acid wash should be performed after every milking, another three (42.9%) were in favor of an acid wash after every fourth caustic wash, and one manager (14.3%) said that one acid wash a day would be optimal.

There was an even greater deviation regarding the average yearly number of occasions when the milking machine was disassembled so that its parts could be cleaned thoroughly, manually. In 29.4% of the farms, this took place every week (52 × a year) and in 17.6% every month (12 × a year). At the same time, 11.8% of the farms never cleaned the milking machines manually (Supplementary Table 7). There was no significant relationship between the number of manual machine cleaning per year and the studied milk production parameters (*p* ≥ 0.1113). The opinions of the interviewed farm managers on the optimal number of manual cleaning sessions per year also greatly varied. Most commonly (28.6% each) a manual cleaning in every 6 months (that is, 2 × a year) and every 3 months (that is, 4 × a year) was considered as optimal, but one manager (14.3%) said that the weekly (52 × ) cleaning would be optimal.

**Figure 4:**
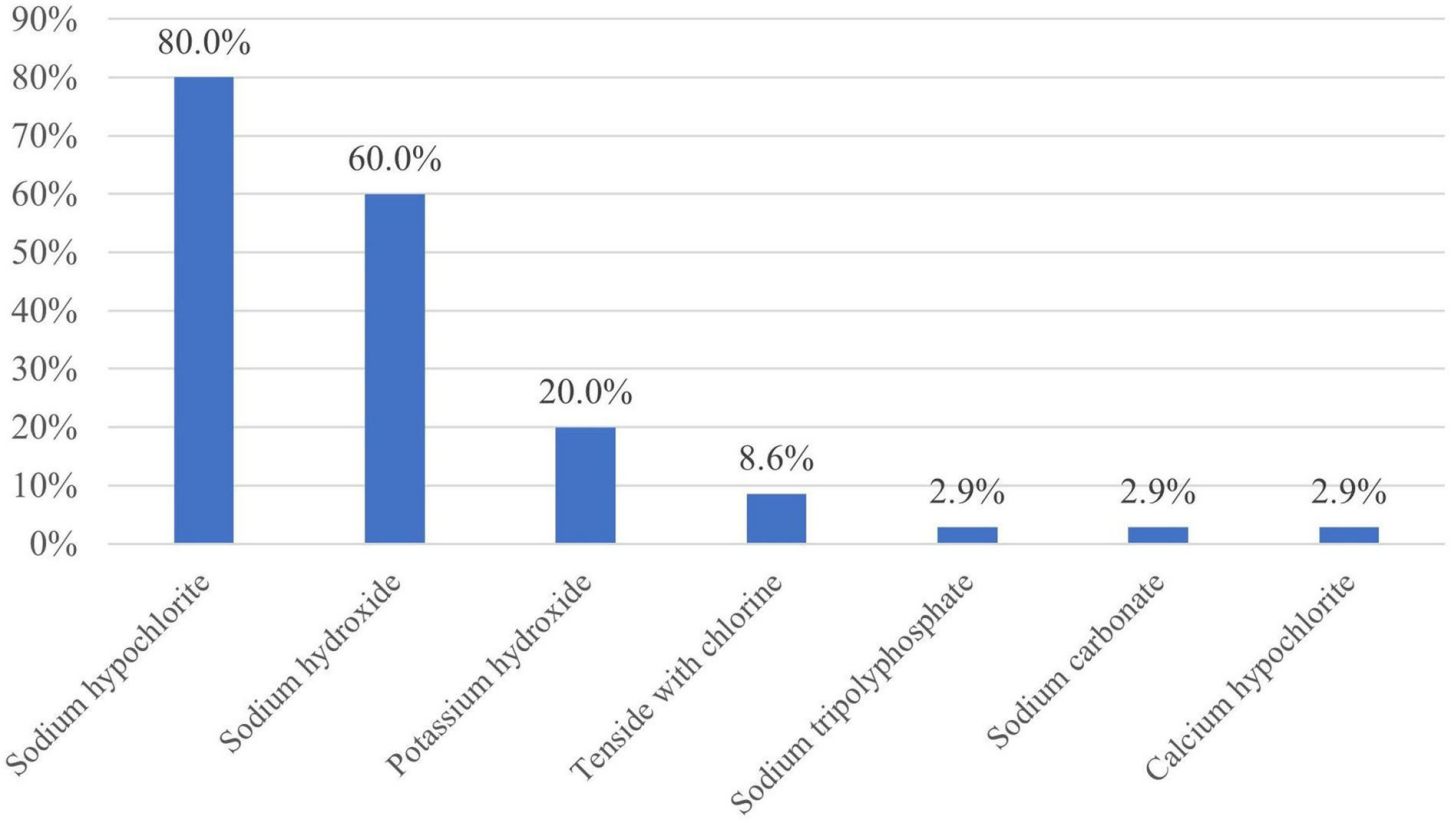
Active ingredients of the caustic detergents (*n* = 35).

Sodium hypochlorite was the most preferred active ingredient of caustic detergents (80.0% of the farms used it), followed by sodium hydroxide (60.0%) and potassium hydroxide (20.0%) (Figure 4). There was no significant relationship between the used caustic detergent and the studied milk production parameters (*p* ≥ 0.426). Three out of seven personally interviewed farm managers (42.9%) were not aware of the active ingredient or the name of the caustic detergent they used on their farm; one (14.3%) could name the product, another (14.3%) could name the distributor, and two of them (28.6%) knew that they used a detergent containing chlorine.

**Figure 5:**
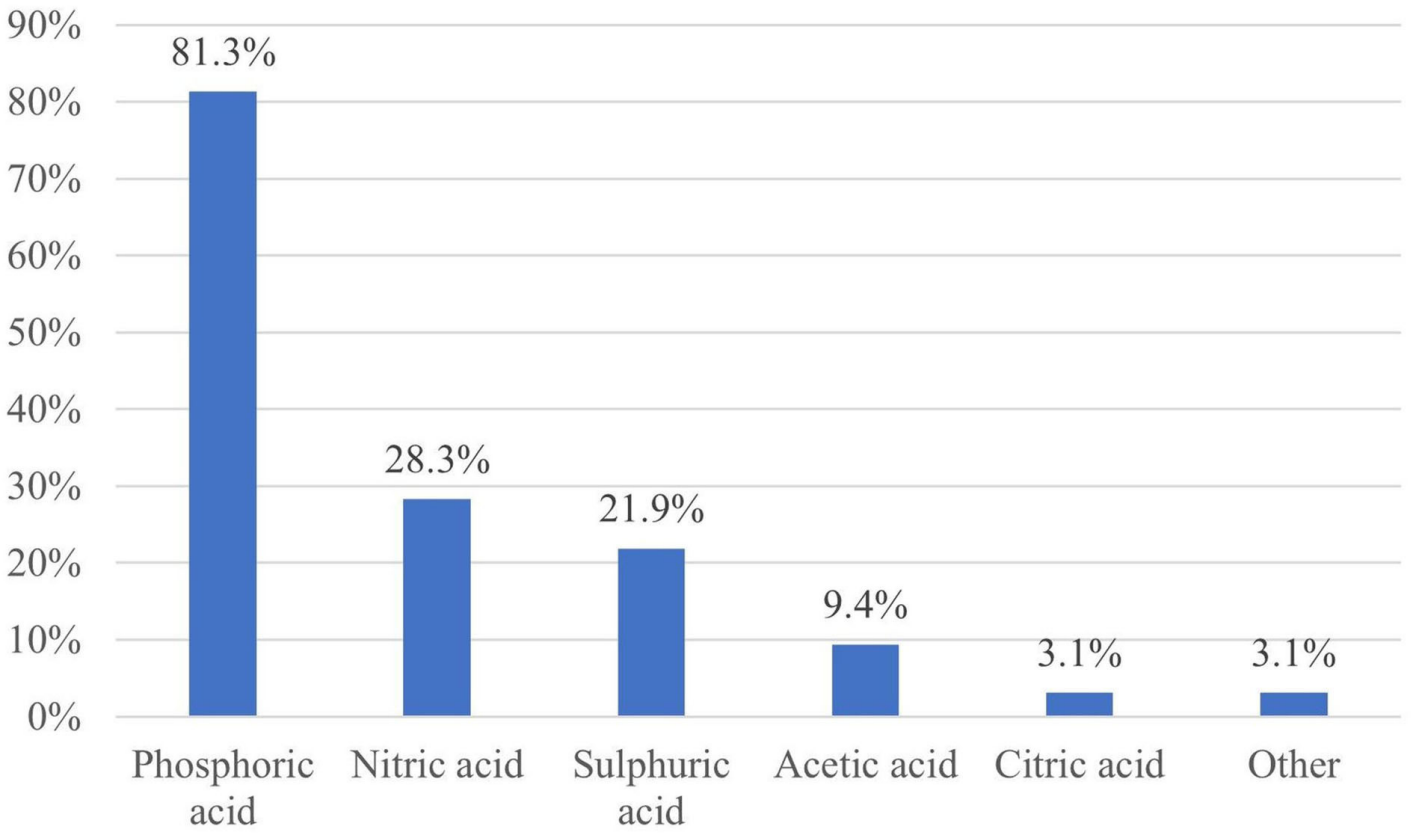
Active ingredients of the acid washes (*n* = 32).

Phosphoric acid was the most used acid (in 81.3% of the farms), followed by nitric acid (in 28.3%), sulfuric acid (in 21.9%), and organic (acetic and citric) acids (in 12.5%) (Figure 5). A significant relationship was found between the use of phosphoric acid combined with nitric acid and the use of a combination of phosphoric acid, nitric acid, and organic acid and SCC (*p* = 0.0483; *p* = 0.0477). The use of phosphoric acid alone tended to associate with SCC (*p* = 0.05698). Only one personally interviewed farm manager (14.3%) had no information regarding the acid cleaning agent, which was used on the farm, three managers (42.9%) knew the name of the distributor, two (28.6%) could name the product but there was only one manager (14.3%), who could name the active ingredient. Thus, it can be concluded that farm managers have only vague information about the active ingredients of the cleaning products used on their farms.

The survey contained questions regarding the concentrations of caustics, acids, and disinfectants. In the surveyed dairies, the most used (38.9%) concentration for caustic solutions was 1% by far, while concentrations of 1.5 and 2% (min. 0.2%; max. 10%) were also frequently used, in 8.3 and 11.1% of the farms, respectively (Supplementary Table 8). The caustic concentration had no significant relationship with any of the studied milk production parameters (*p* ≥ 0.4369). Four out of seven managers (57.1%) did not know the concentration of the caustic solutions, which were used on their farms, the other three (42.9%) said 2–3% concentration, which—based on the results of the questionnaire—was probably greater than the actual values; therefore, they were not aware of the exact numbers. However, all of them stated that they used them according to the product manuals.

The acids were mostly (40.0%) used in a concentration of 1% also, but concentrations of 0.5%; 2% and 5% (min. 0.2%; max. 10%) were also quite common: in 8.6, 11.4, and 8.8% of the farms, respectively (Supplementary Table 9). The acid concentration had no significant relationship with any of the studied milk production parameters (*p* ≥ 0.6543). Five out of seven farm managers (71.4%) were not aware of the concentration of the acid solutions, which were used on their farms, one (14.3%) manager said 0.2% and another 0.3%, which—based on the results of the questionnaire—were probably lower than the actual ones. However, all of them stated that they used the acids according to the product manuals.

Out of 43 surveyed farms, only 17 (39.5%) reported the use of a separate disinfectant for the milking machines. Peracetic acid was the most used disinfectant (88.2%), and 29.4% of the dairies also reported the use of hydrogen peroxide (*n* = 17). The use of peracetic acid was significantly associated with the percentage share of marketed milk (*p* = 0.0432). The results of the personal interviews corresponded with the results of the questionnaires; four farm managers (57.1%) said that they did not use a separate disinfectant while three (42.9%) reported using peracetic acid. The disinfectants were used in concentrations between 0.1 and 10%, but usually (in 61.5% of the farms) between 0.3 and 2% (*n* = 13) (Supplementary Table 10). The concentration of disinfectant had a significant relationship with the percentage share of marketed milk (*p* = 0.0356). In the dairies, where a separate disinfectant was used, none of the farm managers was aware of its concentration, but they all stated that the disinfectants were used according to the product manuals.

**Figure 6:**
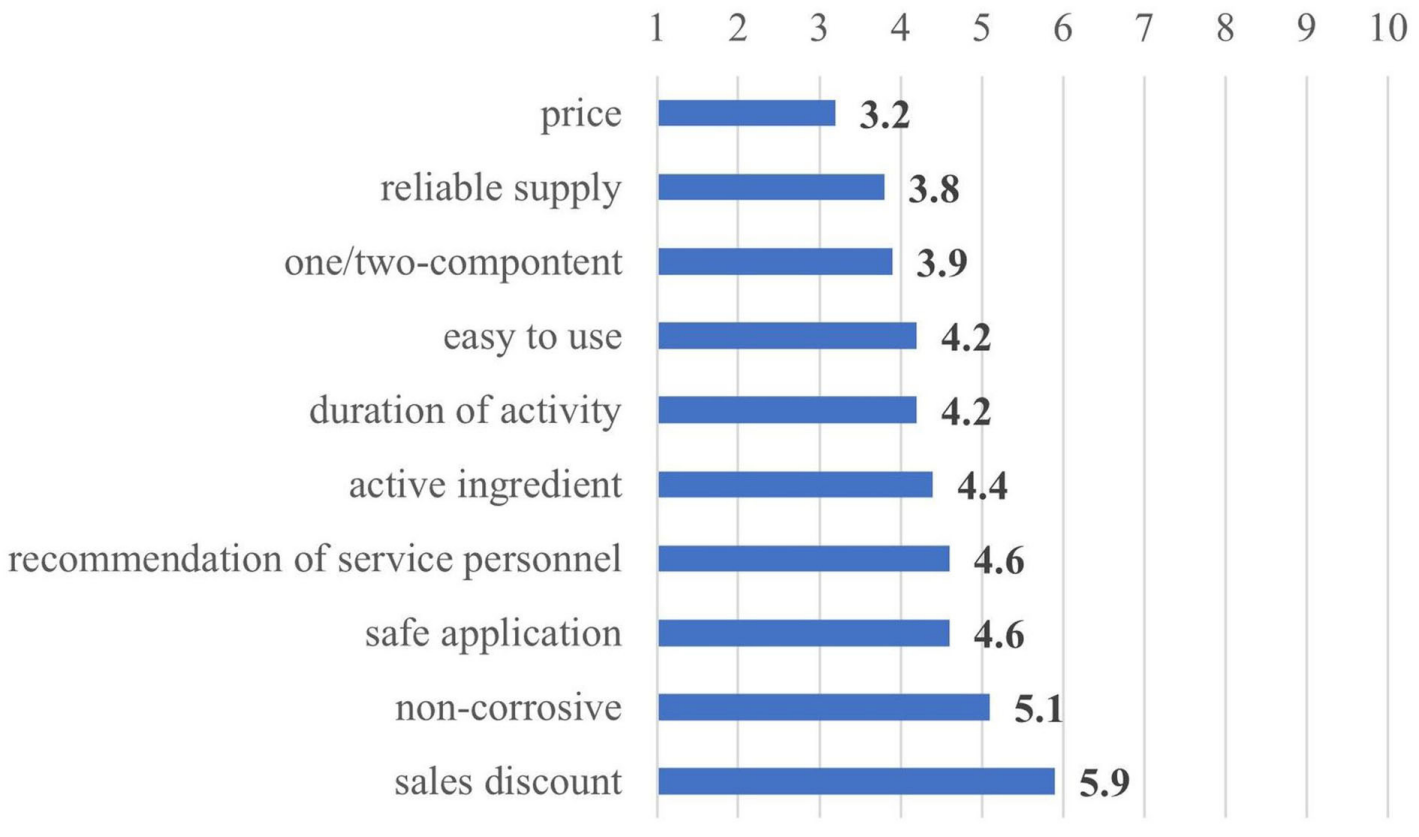
Importance of different procurement factors for milking machine cleaning products (*n* = 38).

The key drivers in the procurement of milking machine cleaning products were also surveyed, and the respondents evaluated the different characteristics of the cleaning products on a scale of 1–10 (“1” for the most important, “10” for the least important characteristic). Figure 6 shows that the price was the most decisive procurement factor for a milking machine cleaning product, followed by reliable supply and the type of the cleaning process (one/two-component). The sale discounts were considered as the least important factor in the procurement process, and the corrosive impact did not prove to be important either.

The results of the personal interviews partly complied with the findings of the survey, because according to the farm managers the most important milking machine cleaning product characteristics were efficacy (all seven managers), followed by good value/price ratio (85.7%), reliable supply (71.4%) and non-corrosive impact (57.1%). Every personally interviewed manager emphasized efficacy, which corresponds to the factors named “active ingredient” and “duration of activity” in the questionnaire and was found to be of moderate importance only in the survey. The reliable distributor, not damaging to teat cups and one/two-component product, was of far smaller importance (28.6% each). Other factors, namely, package size, easy to use, long shelf life, small per-unit cost, safe application, active at low temperatures, and no residues were mentioned once (14.3%) by the farm managers.

## Discussion

### Pre-milking teat disinfection

Effective pre-milking teat sanitation reduces the number of bacteria on teat skin, thus decreasing bacterial contamination of milk and improving milk quality (12, 13). It has been well-established that reducing teat end exposure to microorganisms can result in a reduced incidence of intramammary infection (14). Almost two-thirds of the surveyed farms (62.8%) used disinfectant teat dips prior to milking, but almost one-quarter of the farms (23.3%) still washed the udder with water. Washing with water is only indicated when the udder is very dirty, but it is not recommended as a routine part of udder preparation (15). The udder was wiped with dry paper towels in almost three-quarters of the farms (73.8%), which cannot play a role in spreading infection (16). A teat cleaning procedure that includes wet cleaning followed by manual drying with a paper towel will result in the lowest bacterial counts (4). In situations where herd infection levels are considered high and where the risk of spread of infection is greater, pre-milking disinfection of clean teats followed by teat drying can be beneficial. However, the routine application of pre-milking teat disinfectants in pasture-grazed herds is unlikely to be of benefit where herd SCC is below 200 × 10^3^ cells/mL (17). In general, when cows were housed indoors, the procedure was found to reduce the incidence of new intramammary infection caused by environmental pathogens by >50% (17).

The pre-milking disinfectants containing compounds of chlorine are preferred in two-thirds (64.3%) of the surveyed commercial Holstein-Friesian herds, which complies with the international findings from Europe (16). The use of chlorohexidine digluconate is shown to have a significant efficacy against *Staphylococcus aureus* and *Streptococcus agalactiae* under experimental challenge conditions (17). The second most common pre-milking disinfectant was lactic acid in the surveyed Hungarian dairies (25.0%). The application of probiotic lactic acid bacteria (LAB) is now considered as the best choice for the treatment of many infectious human diseases and the control of bovine mastitis, and the use of probiotic LAB teat disinfectant as a protective barrier to inhibit pathogens and to improve the microbial balance of the teat proved to be beneficial (3). Therefore, the use of lactic acid as a pre-milking disinfectant might be further strengthened in dairy cattle farms to achieve a better microbial balance on the teat skin, thus, have a greater resistance against udder infections.

### Post-milking teat disinfection

According to Oliver et al. (18), almost on every farm iodine is considered as the optimal active ingredient followed by lactic acid in post-milking disinfection, which can largely reduce the incidence of clinical mastitis. These findings correspond to the results of our survey which showed that 53.8% of the surveyed large Holstein-Friesian farms used iodine and 41.0% used lactic acid as active ingredients in their post-milking teat disinfection process, and the use of iodine and of lactic acid combined with other active ingredients resulted in significantly lower SCC. In the United States, iodine-based teat disinfectants are used in 66% of pre-milking and in 84% of post-milking disinfections in large dairy units, and the post-milking teat disinfection with barrier properties and higher free iodine content reduced the risk of clinical mastitis (4). Fitzpatrick et al. (19) stated that the most effective post-milking products to reduce the bacterial load on teat skin contained 0.6% diamine and 0.5% chlorhexidine, 0.6% diamine, and 0.5% w/w iodine in the case of staphylococcal, streptococcal, and coliform isolates, respectively. This is in harmony with other studies where iodine-based products were shown to be effective against a wide range of udder pathogens (20–22), although it should be emphasized that higher iodine concentrations may occur in milk by using these products, which might be an important food safety factor in infant formula production (23).

According to the personally interviewed farms managers, the drying impact on teat skin is an important characteristic of the teat disinfectants that should not dry the skin too much. This is an issue mainly with disinfectants containing iodine, as they can significantly dry the skin and manufacturers try to counterbalance this unwanted effect by using different additives. Chemical disinfectants can reduce the major pathogen infections, but the high concentration of chemical substances raised the concern of potential residues in milk (24). Therefore, during teat disinfections, the farm managers might be encouraged to use products with lower risk to human health. Introducing effective teat disinfectants with chemicals, which occur naturally in milk, is an opportunity in the udder health management, because concerns of residues in milk are minimized (2). Nevertheless, farm conditions and udder health management practices have a significant impact on the effectiveness of teat disinfection (21).

### Milking machine cleaning

The efficacy of milking machine cleaning depends on the working solution content, the temperature of the water, and the application of sanitizer (18). Meanwhile, four out of seven (57.1%) personally interviewed farm managers considered three-phase cleaning systems better than five-phase ones, the five-phase cleaning systems were more common in the Holstein-Friesian dairy units, and it can be stated that the five-phase washing and disinfection are considered equally efficient, and the number of cleaning phases depends mainly on the specifications of the milking equipment manufacturers.

The typical automatic three-phase cleaning process can be divided into three different main phases: pre-rinse, washing phase, and post-rinse. During the washing phase, alkaline or acid detergents should be used. The alkaline detergent helps to remove organic deposits, such as milk protein and fat. Most detergents can work effectively at hot temperatures. Acid detergent is used periodically to remove mineral deposits from water and milk. The frequency of acid washing depends on the hardness of the water used on the farm (7). The number of acid descaling washes per week revealed a very diverse picture in our study: it ranged from once a week to 21 times a week, that is, after every milking, but most commonly it was performed daily or two to three times per week. The use of daily cold caustic cleaning in conjunction with daily hot acid cleaning or cleaning with a hot detergent/sanitizer twice a day maintained the lowest total bacterial count in milk and on plastic surfaces (18).

Sodium hypochlorite was the most preferred active ingredient of caustic detergents, 80.0% of the surveyed commercial dairy farms used it. While the addition of sodium hypochlorite to the pre-milking rinse water may have benefits in sanitizing internal milking equipment surfaces, it may also result in the formation of the residue of tri-chloromethane (TCM) in milk (25). Farms using cleaning products that contain a high sodium hypochlorite content (>8%) are highlighted as being more likely to result in TCM residues in bulk tank milk (26). The international agency for research on cancer states that TCM is possibly carcinogenic to humans (27). In the surveyed Holstein-Friesian dairies, the most used concentration of caustic solutions was 1% by far, while concentrations of 1.5 and 2% were also frequently used. A potential alternative sanitizer for milking machines might be peracetic acid. The addition of peracetic acid in the final rinse water and as a replacement for sodium hypochlorite would also reduce or eliminate TCM residues (5). 39.5% of the surveyed farms reported using a separate disinfectant for the milking machines which was peracetic acid in 88.2%. Peracetic acid was also more effective against *Prototheca zopfii* than sodium hypochlorite or iodine (28). A further potential alternative sanitizer is quaternary ammonium compounds (QAC) that are non-oxidizing surfactant-based disinfectants. Some of these compounds (diethyl ammonium chloride and dimethyl benzyl ammonium chloride) are now being promoted as an alternative to traditional sanitizer products; however, Gleeson et al. (5) found that the QAC product is not suitable for the cleaning of milking equipment due to the foaming effect of the product during the wash circulation.

## Conclusions

A significant share (16.3%) of the surveyed Holstein-Friesian farms did not use any pre-milking disinfectants, which was still a considerable percentage, and applying pre-milking disinfection might improve the udder health in these dairy units. Many products of both pre- and post-milking teat disinfectants are available on the market, but compounds of chlorine are the most preferred active ingredients for pre-milking disinfectants while iodine in post-milking disinfection. Lactic acid is ranked second in both disinfections. The decision-makers on the farms prefer these active ingredients primarily because of their efficacy, which is the most decisive factor in buying teat disinfectants. Price and continuous, reliable supply are also important factors during procurement. Teat disinfectants that are colored, that is, easy to see on the teat skin, and consist of one component are also more preferred by the farm managers. Advertisements and sales discounts have no significant effect on the procurement of teat disinfectants.

The decision-makers on the Holstein-Friesian farms are not as well-informed regarding milking machine cleaning products (caustics and acids) and their use as they are as regards to teat disinfectants. In many cases, they are not aware of their active ingredients and/or concentrations, they only refer to the product manuals. Their opinions greatly differ on that how frequently an acid wash is needed. In some herds (11.9%), acid wash was performed after every milking, but more than half of the farms (52.4%) only run an acid wash every other day or even less frequently. Several milking machine cleaning products, both caustics and acids, are available on the market, but sodium hypochlorite and sodium hydroxide are the most preferred active ingredients of caustic detergents while phosphoric acid is the most used acidic product. Furthermore, 39.5% of the large commercial dairies used a separate milking machine disinfectant, in most cases, peracetic acid. The use of milking machine detergents is greatly influenced by the milking equipment because milking machine distributors often specify which sanitizers are allowed.

According to our knowledge, this was the first scientific, large-scale study assessing the use of teat disinfectants and milking machine cleaning products in Hungarian dairy cattle farms, but the limitation of the survey is the non-representative nature of the sample. As most of the teat disinfectants and milking machine cleaning products proved to be similarly effective in milk production, the use of those, which may pose less risks to human health and the environment, might be more encouraged in the future.

## Data availability statement

The raw data supporting the conclusions of this article will be made available by the authors, without undue reservation.

## Ethics statement

The revised survey was reviewed by the Scientific Research Committee of the Faculty of Veterinary Science, Budapest, and found exempt from human subject protection regulations. The participants provided their written informed consent to participate in this survey.

## Author contributions

LÓ conceived and designed the study, collected the data, and acquired funding. DI developed the statistical models and analyzed the data. DI and LÓ contributed to the conceptualization and writing the paper. All authors contributed to manuscript revision, read and approved the submitted version.

## Funding

This project was supported by the European Union and co-financed by the European Social Fund: (1) EFOP-3.6.1-16-2016-00024 Innovations for Intelligent Specialization on the University of Veterinary Science and the Faculty of Agricultural and Food Sciences of the Széchenyi István University Cooperation and (2) EFOP-3.6.3-VEKOP-16-2017-00005 Strengthening the scientific replacement by supporting the academic workshops and programs of students, developing a mentoring process.

## Conflict of interest

The authors declare that the research was conducted in the absence of any commercial or financial relationships that could be construed as a potential conflict of interest.

## Publisher's note

All claims expressed in this article are solely those of the authors and do not necessarily represent those of their affiliated organizations, or those of the publisher, the editors and the reviewers. Any product that may be evaluated in this article, or claim that may be made by its manufacturer, is not guaranteed or endorsed by the publisher.
