# Supplementary material for: The use of teat disinfectants and milking machine cleaning products in commercial Holstein-Friesian farms
Source: Front Vet Sci. 2022 Oct 19;9:956843. doi: 10.3389/fvets.2022.956843 (PMC9627983; doi:10.3389/fvets.2022.956843)
Supplement: Supplementary file 1 [file Data_Sheet_1.docx]

**Supplementary Table 1.** Pre-milking teat disinfection procedures (n=43)

| **Pre-milking disinfection procedures** | **Number of farms** | **Share (%)** |
| --- | --- | --- |
| Disinfectant teat dips | 28 | 65.1 |
| Disinfectant wash or foaming | 6 | 14.0 |
| Impregnated papers | 2 | 4.7 |
| No pre-milking disinfection | 7 | 16.3 |

**Supplementary Table 2.** Component number of pre-milking teat disinfectants (n=36)

| **Component number** | **Number of farms** | **Share (%)** |
| --- | --- | --- |
| One-component | 26 | 72.2 |
| Two-component | 5 | 13.9 |
| One- and two-component | 3 | 8.3 |
| Impregnated paper | 2 | 5.6 |

**Supplementary Table 3.** Component number of post-milking teat disinfectants (n=40)

| **Component number** | **Number of farms** | **Share (%)** |
| --- | --- | --- |
| One-component | 27 | 67.5 |
| Two-component | 11 | 27.5 |
| One- and two-component | 2 | 5.0 |

**Supplementary Table 4.** Type of milking machine cleaning systems (n=42)

| **Cleaning system** | **Number of farms** | **Share (%)** |
| --- | --- | --- |
| 5-phase | 25 | 59.5 |
| 3-phase | 15 | 35.7 |
| Both | 2 | 4.8 |

**Supplementary Table 5.** Number of daily milking machine cleanings (n=42)

| **Number of daily cleanings** | **Number of farms** | **Share (%)** |
| --- | --- | --- |
| 1x | 2 | 4.8% |
| 2x | 31 | 73.8% |
| 3x | 9 | 21.4% |

**Supplementary Table 6.** Number of acid descaling washes per week (n=42)

| **Number of weekly acid descaling washes** | **Number of farms** | **Share (%)** |
| --- | --- | --- |
| 1x | 5 | 11.9 |
| 2x | 10 | 23.8 |
| 3x | 7 | 16.7 |
| 3.5x | 1 | 2.4 |
| 7x | 11 | 26.2 |
| 14x | 3 | 7.1 |
| 21x | 5 | 11.9 |

**Supplementary Table 7.** Yearly number of manual milking machine cleanings (n=34)

| **Number of manual cleanings** | **Number of farms** | **Share (%)** |
| --- | --- | --- |
| 0 | 4 | 11.8 |
| 2x | 3 | 8.8 |
| 3x | 2 | 5.9 |
| 4x | 3 | 8.8 |
| 5x | 3 | 8.8 |
| 6x | 1 | 2.9 |
| 12x | 6 | 17.6 |
| 24x | 1 | 2.9 |
| 36x | 1 | 2.9 |
| 52x | 10 | 29.4 |

**Supplementary Table 8.** Concentration of the caustic detergents (n=36)

| **Caustic concentration** | **Number of farms** | **Share (%)** |
| --- | --- | --- |
| 0.2% | 1 | 2.8 |
| 0.5% | 2 | 5.6 |
| 0.65% | 1 | 2.8 |
| 0.7% | 1 | 2.8 |
| 0.75% | 2 | 5.6 |
| 1.0% | 14 | 38.9 |
| 1.5% | 3 | 8.3 |
| 2.0% | 4 | 11.1 |
| 2.5% | 2 | 5.6 |
| 3.0% | 1 | 2.8 |
| 4.0% | 1 | 2.8 |
| 5.0% | 2 | 5.6 |
| 10.0% | 2 | 5.6 |

**Supplementary Table 9.** Concentration of the acid detergents (n=35)

| **Acid concentration** | **Number of farms** | **Share (%)** |
| --- | --- | --- |
| 0.2% | 1 | 2.9 |
| 0.5% | 3 | 8.6 |
| 0.65% | 1 | 2.9 |
| 0.7% | 1 | 2.9 |
| 0.75% | 2 | 5.7 |
| 1.0% | 14 | 40.0 |
| 1.5% | 2 | 5.7 |
| 2.0% | 4 | 11.4 |
| 2.5% | 1 | 2.9 |
| 4.0% | 1 | 2.9 |
| 5.0% | 3 | 8.6 |
| 10.0% | 2 | 5.7 |

**Supplementary Table 10.** Concentration of disinfectants in milking machine cleanings (n=13)

| **Disinfectant concentration** | **Number of farms** | **Share (%)** |
| --- | --- | --- |
| 0.1% | 1 | 7.7 |
| 0.2% | 1 | 7.7 |
| 0.3% | 2 | 15.4 |
| 0.5% | 2 | 15.4 |
| 1.0% | 2 | 15.4 |
| 2.0% | 2 | 15.4 |
| 2.5% | 1 | 7.7 |
| 10.0% | 2 | 15.4 |
